# Cochlear Synaptopathy due to Mutations in *OTOF* Gene May Result in Stable Mild Hearing Loss and Severe Impairment of Speech Perception

**DOI:** 10.1097/AUD.0000000000001052

**Published:** 2021-04-27

**Authors:** Rosamaria Santarelli, Pietro Scimemi, Marco Costantini, María Domínguez-Ruiz, Montserrat Rodríguez-Ballesteros, Ignacio del Castillo

**Affiliations:** 1 Department of Neurosciences, University of Padua, Padua, Italy; 2 Audiology Service, Santi Giovanni e Paolo Hospital, Venezia, Italy; 3 Servicio de Genética, Hospital Universitario Ramón y Cajal, Madrid, Spain; 4 Centro de Investigación Biomédica en Red de Enfermedades Raras (CIBERER), Madrid, Spain.

**Keywords:** Auditory neuropathy, Cochlear implants, Electrocochleography, *OTOF*-related hearing loss, Psychoacoustical tuning curves, Speech perception

## Abstract

Congenital profound hearing loss with preserved cochlear outer hair cell activity (otoacoustic emissions and cochlear microphonic) is the most common phenotype associated with mutations in the *OTOF* gene. The aim of this study was to investigate the pathophysiological mechanisms behind the auditory dysfunction in five patients (2 adults and 3 children) carrying biallelic mutations in *OTOF*, who showed an uncommon phenotype of mild hearing impairment associated with severe difficulties in speech perception and delay of language development.

**Design:**

Patients underwent audiometric assessment with pure-tone and speech perception evaluation, and otoacoustic emissions and auditory brainstem response recording. Cochlear potentials were recorded in all subjects through transtympanic electrocochleography in response to clicks delivered in the free field from 120 to 60 dB peak equivalent SPL and were compared to recordings obtained from 20 normally hearing controls and from eight children with profound deafness due to mutations in the *OTOF* gene. Three patients out of five underwent unilateral cochlear implantation. Speech perception measures and electrically evoked auditory nerve potentials were obtained within 1 year of cochlear implant use.

**Results:**

Pathogenic mutations in the two alleles of *OTOF* were found in all five patients, and five novel mutations were identified. Hearing thresholds indicated mild hearing loss in four patients and moderate hearing loss in one. Distortion product otoacoustic emissions were recorded in all subjects, whereas auditory brainstem responses were absent in all but two patients, who showed a delayed wave V in one ear. In electrocochleography recordings, cochlear microphonics and summating potentials showed normal latency and peak amplitude, consistently with preservation of both outer and inner hair cell activity. In contrast, the neural compound action potential recorded in normally hearing controls was replaced by a prolonged, low-amplitude negative response. No differences in cochlear potentials were found between OTOF subjects showing mild or profound hearing loss. Electrical stimulation through the cochlear implant improved speech perception and restored synchronized auditory nerve responses in all cochlear implant recipients.

**Conclusions:**

These findings indicate that disordered synchrony in auditory fiber activity underlies the impairment of speech perception in patients carrying biallelic mutations in *OTOF* gene who show a stable phenotype of mild hearing loss. Abnormal nerve synchrony with preservation of hearing sensitivity is consistent with selective impairment of vesicle replenishment at the ribbon synapses with relative preservation of synaptic exocytosis. Cochlear implants are effective in restoring speech perception and synchronous activation of the auditory pathway by directly stimulating auditory fibers.

## INTRODUCTION

Auditory neuropathy is a hearing disorder characterized by disruption of the temporal coding of acoustic signals in the auditory nerve fibers affecting auditory perceptions dependent on the processing of temporal cues, the magnitude of these perceptual changes being independent of the changes in audibility (Starr et al. 2008 for a review). The mechanisms believed to be involved are functional alterations at presynaptic and postsynaptic sites, including inner hair cell (IHC) depolarization, neurotransmitter release from ribbon synapses (cochlear synaptopathy), spike initiation in auditory nerve terminals, loss of nerve fibers, and the neural dys-synchrony accompanying demyelination.

The most well-known form of cochlear synaptopathy is associated with mutations in the *OTOF* gene (DFNB9) encoding otoferlin, a transmembrane protein involved in glutamate neurotransmitter release at the IHCs ribbon synapses. Specifically, otoferlin might have a dual role in synaptic vesicle exocytosis, as a calcium sensor for synaptic vesicle fusion (Roux et al. 2006), and as a priming factor enabling fast vesicle replenishment (Pangršič et al. 2010).

The human *OTOF* gene contains 48 exons and codes for multiple long and short isoforms of the protein, by alternative splicing and by using several translation initiation sites (Yasunaga et al. 2000). Otoferlin belongs to the ferlin family of proteins, which contain several repeats of the C2 domain, a calcium-binding structural module. Otoferlin long isoforms have six C2 domains (C2A to C2F), whereas the short isoforms have only three C2 domains (C2D to C2F). All isoforms are anchored to membrane through a transmembrane domain that is located in the C-terminal part of the protein (Yasunaga et al. 2000).

Mutations in *OTOF* account for 1.4 to 5% of cases of autosomal recessive nonsyndromic hearing impairment in different populations (Rodríguez-Ballesteros et al. 2008). Most patients present with a very homogeneous phenotype of severe to profound congenital hearing loss. More than 50% of children carrying two mutant alleles of *OTOF* gene have preserved outer hair cell (OHC) function as indicated by cochlear microphonic (CM) and otoacoustic emissions (OAEs) recordings.

In our previous studies, recordings of cochlear potentials through transtympanic electrocochleography (ECochG) in profoundly deaf children carrying two mutant alleles in *OTOF* indicated preservation of both outer and IHC function (Santarelli et al. 2009, 2015). In contrast, the compound action potential (CAP), resulting from synchronous activation of auditory fibers, was replaced by a prolonged low-amplitude negative potential, consistently with abnormal activation of auditory fibers due to impaired multivesicular release with consequent alterations of spike generation. Cochlear implants proved to be effective in restoring hearing sensitivity and speech perception in these subjects (Santarelli et al. 2015).

Moreover, some missense mutations in *OTOF* have been reported in patients showing a temperature-sensitive phenotype (Varga et al. 2006; Romanos et al. 2009; Marlin et al. 2010; Wang et al. 2010; Matsunaga et al. 2012; Zhang et al. 2016), while few have been associated with progressive hearing loss (Chiu et al. 2010; Yildirim-Baylan et al. 2014; Fedick et al. 2016). Specifically, in the temperature-sensitive phenotype afebrile patients have normal or only mildly elevated hearing thresholds, while speech perception is almost normal in quiet environments, appearing abnormally reduced only in the presence of background noise (Starr et al. 1998; Varga et al. 2006). When the core temperature rises, hearing thresholds increase, leading to variable degrees of hearing loss, and speech perception worsens remarkably, regardless of the extent of the individual’s hearing threshold elevation (Starr et al. 1998; Varga et al. 2006; Marlin et al. 2010; Matsunaga et al. 2012).

In this study, we investigated the pathophysiological mechanisms behind the auditory dysfunction identified in five patients carrying biallelic mutations in the *OTOF* gene who showed a distinctive, uncommon phenotype, namely stable mild hearing loss associated with severe impairment of speech perception and delay in language development. None showed the temperature-sensitive phenotype. Audiological and electrophysiological findings indicated abnormal activation of auditory fibers with disruption of temporal coding of acoustic input. The use of a cochlear implant was effective in restoring speech perception and synchronous activation of auditory fibers.

## MATERIALS AND METHODS

### Subjects

**Fig. 1.**
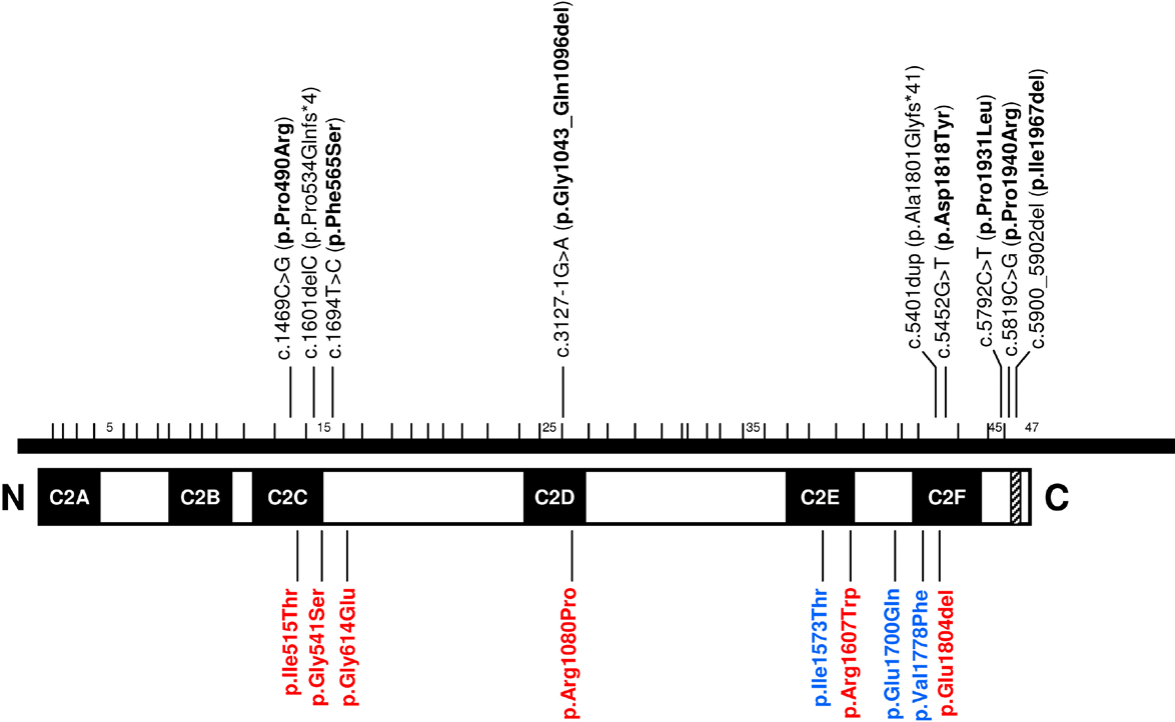
Mutations in the *OTOF* gene causing mild phenotypes. A horizontal bar depicts the cDNA, small vertical bars delimiting the exons, which are numbered. The protein is represented by a rectangle, boxes indicating the predicted domains of the protein. Upper part, mutations that were found in this study; lower part, mutations in the literature. Nontruncating mutations (missense mutations, in-frame deletions) are shown in bold. Mutations related to the temperature-sensitive phenotype are shown in red. Mutations causing a progressive hearing impairment are shown in blue. Vertical lines indicate the position of each mutation on the reference cDNA (accession number NM_001287489.1) and on the correlated position in the protein. Black boxes, C2 domains. Cross-hatched box, transmembrane domain.

We investigated the hearing function in five subjects carrying biallelic mutations in the *OTOF* gene (one male; age range 2 to 27 years). Details of their genetic and audiological findings are summarized in Table 1, while the location of their pathogenic mutations is shown in Figure 1.

**TABLE 1.** Genetic and audiological findings from OTOF subjects with mild to moderate hearing loss

Subjects No.	1	2	3	4	5
Gender	F	F	F	M	F
Age tested	22 yrs	27 yrs	5 yrs (72 mo)	2 yrs (18 mo)	3 yrs (36 mo)
Genotype	c.3127-1G>A/c.1469C>G	c.5819C>G/c.5819C>G	c.5452G>T/c.5792C>T	c.5900_5902del/c.5401dup	c.1601delC/c.1694T>C
Audiology R/L					
Hearing loss	Mild/Mild	Mild/Mild	Mild/Mild	Mild	Moderate
PTA (dB)	25/20	40/36	26/21	27	51
Acoustic Reflexes*	+/+	+/+	ABS/ABS	+/+	ABS/ABS
OAEs	+/+	+/+	+/+	+/+	+/+
ABRs	ABS/+	ABS/ABS	ABS/+	ABS/ABS	ABS/ABS
Cochlear implantation					
Cochlear implant	—	CI24RECA	CI512	—	CI512
Implanted ear	—	Right	Right	—	Right
Age at implant	—	28 yrs	6 yrs (76 mo)	—	4 yrs (41 mo)
Aided thresholds (dB)	—	26	24	—	35
Disyllable recognition	55%	72%	52%	—	—
Disyllable recognition after CI	—	88%	95%	—	90%
Sentence recognition	52%	60%	—	—	—
Sentence recognition after CI	—	95%	98%	—	78%
Consonant identification	43%	28%	—	—	—
Consonant identification after CI	—	72%	79%	—	78%

+, present; ABRs, auditory brainstem responses; ABS, absent; Aided threshold, average thresholds at 0.25, 0.5, 1, 2, and 4 kHz as measured in the free field with subjects wearing the sound processor; CI, cochlear implantation; OAEs, otoacoustic emissions; PTA, pure-tone average (average thresholds at 0.5, 1, 2, 4 kHz); R/L, right ear/left ear.

* Measured ipsilaterally to the stimulated ear.

Three patients were evaluated as children (#3 to #5). They had passed the newborn hearing screening by OAEs recording and were referred for assessment because of delay in language development. Findings from subject #4 have been partially reported in a previous study (Vogl et al. 2016).

Two subjects were diagnosed in their adult life. Subject #1 was evaluated at another institution at the age of 2 because of delay in speech development, but, since hearing thresholds were normal, she was referred for cognitive assessment. When we saw her, she was able to speak quite fluently, but speech perception relied almost completely on lipreading. Subject #2 had undergone several audiological assessments since childhood, first for delay in language development and thereafter for impairment of speech perception. At the time of evaluation, language was quite good, but verbal production showed several phonological and morphosyntactic errors, while speech perception relied on lipreading. Both adult subjects had tried hearing aids without benefit.

None showed the temperature-sensitive phenotype.

CT and MRI scans of the head and ear as well as growth and motor development were normal in all.

Three patients (#2, #3, #5) received unilateral cochlear implant, whereas two refused the procedure.

### Genetic Analysis

DNA was extracted from peripheral blood samples by using an automated system (Chemagic MSM I; Chemagen, Baesweiler, Germany). Sanger sequencing was used to screen for mutations in *OTOF*, as reported (Rodríguez-Ballesteros et al. 2008).

Mutation nomenclature is based on GenBank accession number NM_001287489.1, which corresponds to the cDNA sequence of the long cochlear *OTOF* isoform. Nomenclature follows current Human Genome Variation Society rules as implemented by the Mutalyzer 2.0.3 program (https://www.mutalyzer.nl/, Leiden University Medical Center, Leiden, The Netherlands). Software for prediction of mutation pathogenicity is indicated in the footnote to Table 2.

**TABLE 2.** Minor allele frequencies and scores of pathogenicity from different predictors for the novel missense mutations that are reported in this study

Mutation	MAF	SIFT	PolyPhen-2	MutationTaster	FATHMM-MKL	PROVEAN	DANN	REVEL
c.1694T>C (p.Phe565Ser)	1.5 × 10^−5^	Damaging (0.001)	Probably damaging (0.999)	Disease causing (1)	Damaging (0.9921)	Damaging (−6.63)	Damaging (0.9988)	Pathogenic (0.9269)
c.5452G>T (p.Asp1818Tyr)	Not reported	Damaging (0.001)	Probably damaging (0.999)	Disease causing (1)	Damaging (0.9419)	Damaging (−7.97, −7.57)	Damaging (0.995)	Pathogenic (0.8949)
c.5792C>T (p.Pro1931Leu)	Not reported	Damaging (0)	Probably damaging (1.000)	Disease causing (1)	Damaging (0.9704)	Damaging (−9.36, −9.25)	Damaging (0.999)	Pathogenic (0.787)
c.5819C>G (p.Pro1940Arg)	<10^−5^	Damaging (0.001)	Probably damaging (0.998)	Disease causing (1)	Damaging (0.9834)	Damaging (−8.61, −8.71)	Damaging (0.9982)	Pathogenic (0.791)

DANN, deleterious annotation using neural networks (https://cbcl.ics.uci.edu/public_data/DANN/); FATHMM-MKL, functional analysis through hidden Markov models (http://fathmm.biocompute.org.uk/fathmmMKL.htm); MAF, minor allele frequency, as provided by Ensembl (https://www.ensembl.org/); PolyPhen-2, polymorphism phenotyping v2 (http://genetics.bwh.harvard.edu/pph2/); MutationTaster (http://www.mutationtaster.org/); PROVEAN, protein variation effect analyzer (http://provean.jcvi.org/index.php); REVEL, rare exome variant ensemble learner (https://sites.google.com/site/revelgenomics/); SIFT, sorting intolerant from tolerant (https://sift.bii.a-star.edu.sg/).

This study was approved by the Ethical Committee of Hospital Universitario Ramón y Cajal (in accordance with the 1964 Declaration of Helsinki). Written informed consent was obtained from all participating subjects, or from their parents in the case of minors.

### Audiological Studies

All audiological tests were performed in a sound-attenuating room (Marvinacustica). In cooperative subjects (#1 to #3), air and bone conduction hearing thresholds were obtained in each ear through conventional audiometry (Madsen Astera 2), whereas the two younger children performed visual reinforcement audiometry in the free field. The degree of hearing impairment was defined by the pure-tone average (PTA) at 0.5, 1, 2, and 4 kHz (Martini et al. 1997).

Acoustic reflex thresholds were measured ipsilaterally and contralaterally to the stimulated ear at frequencies of 0.5, 1, 2, and 4 kHz. (Madsen Zodiac impedance audiometer). They were considered absent when no response was found at intensities higher than 110 dB HL.

Articulation gain curves were obtained using disyllabic, phonetically balanced words from 10 Italian wordlists for adults or children, each including 20 items (Cutugno et al. 2000). Ten words were presented for each stimulus intensity.

Distortion product otoacoustic emissions (DPOAEs) were obtained using the ILO-292 OAE system. Primary tones were presented at 65/55 dB SPL and the f2/f1 ratio was kept at 1.21. The f2 frequency was increased in 1/4 octave steps from 1.0 kHz. The upper f2 frequency was kept at 8.0 kHz in subjects #1, 2 and #4, and at 10.0 and 6.0 kHz in subjects #3 and #5, respectively.

### Psychoacoustic Studies

Gap detection was measured in the two adult subjects (#1 and #2). Stimuli were presented in the free field through one loudspeaker placed 1 m away from the front of the subject’s head at an intensity of 30 dB above threshold. We used the procedures reported by Zeng et al. (2005). Briefly, gap detection was evaluated by inserting a silent interval in the center of broadband white noise (20 to 14 000 Hz). The stimulus paradigm consisted of a three-interval, three-alternative, forced-choice, two-down and one-up procedure to fulfill the 70.7% percent correct response criterion. White noise was calibrated in the free field by means of a Brüel and Kjaer 4165 microphone (mounted on the 800 B Larson–Davis sound level meter).

Psychophysical tuning curves (PTCs) were measured in subject #2 for each ear at frequencies of 1, 2, and 4 kHz by presenting the sinusoidal signal at a fixed sensation level (15 dB SL) together with a narrowband noise used as the masker. The level of masker required to just mask the signal was determined for 19 masker center frequencies. The stimulus paradigm consisted of a two-alternative, forced-choice, two-up and one-down procedure. The masker was presented in two intervals (duration 200 ms, interstimulus interval 500 ms), one of which (chosen at random) contained the signal (duration 200 ms). The masker level was changed with a step size of 4 dB until the level of the masker barely masked the signal. Thereafter, the two-up and one-down procedure was repeated using a step size of 1 dB. The measurements were made using a Tucker–Davis Technologies system controlled by a computer. The masker and signal were generated according to Kluk and Moore (2004).

### Speech Perception Tests

In the two adult subjects, the recognition of disyllabic words and identification of consonants were evaluated by presenting recorded speech stimuli in the free field through one loudspeaker placed 1 m away from the front of the subject’s head. Speech material comprised digital anechoic recordings of a native Italian female speaker. Tests were administered at 70 dB(A) in quiet. In subject #2, the recognition of disyllabic words was also performed in the presence of competing speech noise at three signal-to-noise (S/N) ratios (+10, +5, and 0) through two additional loudspeakers placed laterally at an angle of 90° on either side of the subject’s head at a distance of 1 m. Sentence recognition was evaluated through live-voice presentation. The speech material was obtained from the protocol of patient candidacy for cochlear implantation (CI) for the Italian language for adult patients (Quaranta et al. 1996). The recognition of disyllabic words was evaluated by means of one of 10 randomly chosen lists each including 20 items. The lists of words were phonetically balanced, so that their frequency was similar to that represented in normal conversation in the Italian language. For the sentence recognition task, subjects were randomly presented with one of three lists of 20 items each.

In consonant identification test, stimuli consisted of confusion matrices compiled from two presentations, each comprising the 16 consonants, b d f g k l m n p r s t v z j t∫ presented in an “a-consonant-a” context (chance 6%).

In children, all tests were administered through live-voice presentation. The speech material was obtained from the protocol of evaluation of speech perception for the Italian language (De Filippis et al. 1997). The speech material was appropriate for the age of 4 to 5 years. The recognition of disyllabic words was evaluated by means of two of 10 randomly chosen lists each including 10 items. For the sentence recognition task, children were randomly presented with one of 10 lists of 10 items each.

### Electrophysiological Studies

Auditory brainstem responses (ABRs) were recorded from scalp electrodes in response to 2000 trials of alternating polarity clicks presented monaurally at a maximum intensity of 125 dB peak equivalent (p.e.) SPL (corresponding to 90 dB nHL relative to the psychoacoustic threshold of normally hearing subjects). The filter settings of the amplifier were set between 5 and 4000 Hz.

The adult patients and children’s parents signed an informed consent form for submission to ECochG recording, which is part of our standard assessment protocol in patients showing the profile of auditory neuropathy. The ECochG protocol was assessed by the regional body for quality control of clinical and therapeutic procedures (CCHSA, Veneto Region 2007–2010).

Adults were tested under local anesthesia and children under general anesthesia. ECochG procedures have been reported elsewhere (Santarelli & Arslan 2013). Briefly, a sterile stainless steel needle electrode was passed through the tympanic membrane and placed on the promontory wall with the aid of an operating microscope. Stimuli consisted of 0.1-ms rarefaction and condensation clicks, delivered separately in the free field by means of two high-frequency drivers (Electro-Voice DH1A/2MT 16 Ω) mounted on a single polyurethane horn (Electro-Voice HP420) with a maximum intensity of 120 dB p.e. SPL (corresponding to 90 dB nHL relative to the psychoacoustic threshold of normally hearing subjects). The stimulus was calibrated in the free field by means of a Brüel and Kjaer 4165 microphone (mounted on an 800 B Larson–Davis sound level meter) placed at 1 m from the base of the polyurethane horn, which corresponded to the distance of the patient’s ear from the horn. The procedure of comparing the peak to peak amplitude of the click to the peak to peak amplitude of a 2-kHz tone was utilized to calibrate the click level (p.e. SPL) (Santarelli et al. 2008).

The stimulus paradigm consisted of an initial click, followed 15 ms later by 10 clicks with an interstimulus interval of 2.9 ms, and the sequence was repeated every 191 ms (Santarelli et al. 2008).

The potentials were differentially amplified (×50,000), filtered (5 to 8000 Hz) and digitized (25 µs) for averaging (500 trials). The procedure of averaging the responses evoked separately by condensation and rarefaction clicks was applied to cancel the CM and extract the CAP with the superimposed summating potential (SP). The resulting curve was subtracted from the potential evoked by condensation clicks to obtain the CM. Since CM attenuation was often incomplete at a high stimulus intensity and CM spectral energy was at a maximum between 1500 and 3000 Hz, a low-pass digital filter (12 dB/octave, cutoff frequency 2000 Hz) was used to attenuate the residual CM, where needed (Santarelli et al. 2008).

Latency was defined relative to CM onset in milliseconds (ms). Amplitude was computed relative to the period 1 ms before CM onset in microvolt (µV).

Cochlear potentials recorded from OTOF patients were compared to the ECochG data previously collected from two groups. The first group included 20 children (age range 3.5 to 6.5 years) tested for presumed cochlear hearing loss, who showed normal thresholds when evoking neural and receptor potentials (Santarelli & Arslan 2013), while the second group comprised eight children with *OTOF*-related profound hearing loss whose ECochG findings have been reported in a previous paper (Santarelli et al. 2015).

Electrically evoked CAPs (e-CAPs) were obtained through the cochlear implant in response to trains of alternate polarity, biphasic, 25 µs width per phase pulses presented at 80 Hz. The evoked electrical activity was recorded two electrodes apart.

### Statistical Analysis

Separate two-factor ANOVAs for repeated measures with factors of group and stimulus intensity were used to evaluate ECochG measures. Post-hoc tests for multiple comparisons were conducted using the Tukey–Kramer procedure. The level of significance was *p* <0.05.

## RESULTS

### Genetic Findings

We studied five Italian subjects who showed mild to moderate sensorineural hearing loss associated with severe impairment of speech perception. Parents of all subjects had normal hearing, indicating compatibility with an autosomal recessive pattern of inheritance. Because of their phenotype, we started by screening the *OTOF* gene. Mutations in the two alleles of *OTOF* were found in all five patients (Table 1). Subject #1 was compound heterozygous for a mutation affecting a splice acceptor site (c.3127-1G>A) and a previously reported missense mutation (c.1469C>G, p.Pro490Arg) (Al-Wardy et al. 2016). Her parents were heterozygous carriers (father c.3127-1G>A; mother c.1469C>G). Subject #2 was homozygous for c.5819C>G (p.Pro1940Arg), and her parents were heterozygous carriers of this mutation. Subject #3 was compound heterozygous for two missense mutations, c.5452G>T (p.Asp1818Tyr) and c.5792C>T (p.Pro1931Leu). Her parents were heterozygous carriers (father c.5792C>T; mother c.5452G>T). Subject #5 was compound heterozygous for the previously reported c.1601delC (p.Pro534Glnfs*4) mutation (Santarelli et al. 2015) and the missense mutation c.1694T>C (p.Phe565Ser). Her parents were heterozygous carriers (father c.1601delC; mother c.1694T>C). In addition, a previous study revealed that subject #4 was compound heterozygous for c.5900_5902del (p.Ile1967del) and c.5401dup (p.Ala1801Glyfs*41) (Vogl et al. 2016).

**Fig. 2.**
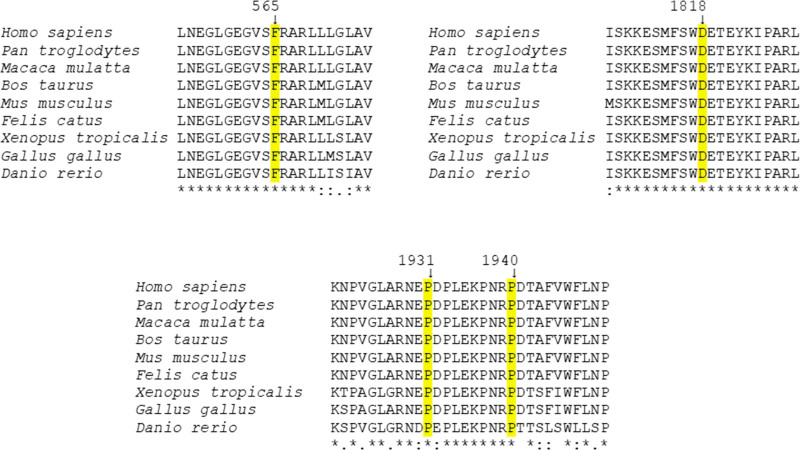
Multiple alignment of sequences stretches of otoferlin proteins to show conservation of the residues affected by the novel missense mutations across diverse vertebrate species. Residues affected by the mutations are indicated. Sequence accession numbers: *Homo sapiens*, NP_001274418.1; *Pan troglodytes*, XP_016803685.1; *Macaca mulata*, XP_014967379.2; *Bos taurus*, NP_001137579.1; *Mus musculus*, NP_001093865.1; *Felis catus*, XP_019683349.1; *Xenopus tropicalis*, XP_031757741.1; *Gallus gallus*, XP_015140684.1; *Danio rerio*, XP_009293007.1. Asterisk, fully conserved residue; two dots, strong conservation; one dot, weaker conservation. Sequences were aligned by using ClustalW (https://www.genome.jp/tools-bin/clustalw).

In total, we report five novel mutations, including one splice-site variant and four missense mutations. Mutation c.3127-1G>A is predicted to result in exon 26 skipping, which would generate an in-frame deletion of 54 residues, which removes the C-terminal part of the C2D domain (p.Gly1043_Gln1096del). All four novel missense mutations affect highly conserved residues in otoferlin across diverse vertebrate species (Fig. 2). Mutation p.Asp1818Tyr affects a residue within the C2F domain, whereas p.Phe565Ser affects a residue close to the C2C domain (Fig. 1). The two other mutations (p.Pro1931Leu and p.Pro1940Arg) lie within a region between the C2F domain and the transmembrane domain, which is highly conserved in proteins of the ferlin family. All four missense mutations were considered to be pathogenic by different predictors (Table 2). Allele frequencies were very low for all five novel variants: either they were not present in databases (c.3127-1G>A, p.Pro1931Leu, p.Asp1818Tyr) or their highest population MAF was extremely low (p.Phe565Ser, p.Pro1940Arg) (Table 2). We concluded that the patients’ genotypes were causative of their hearing impairments.

### Hearing Thresholds, DPOAEs, and ABRs

**Fig. 3.**
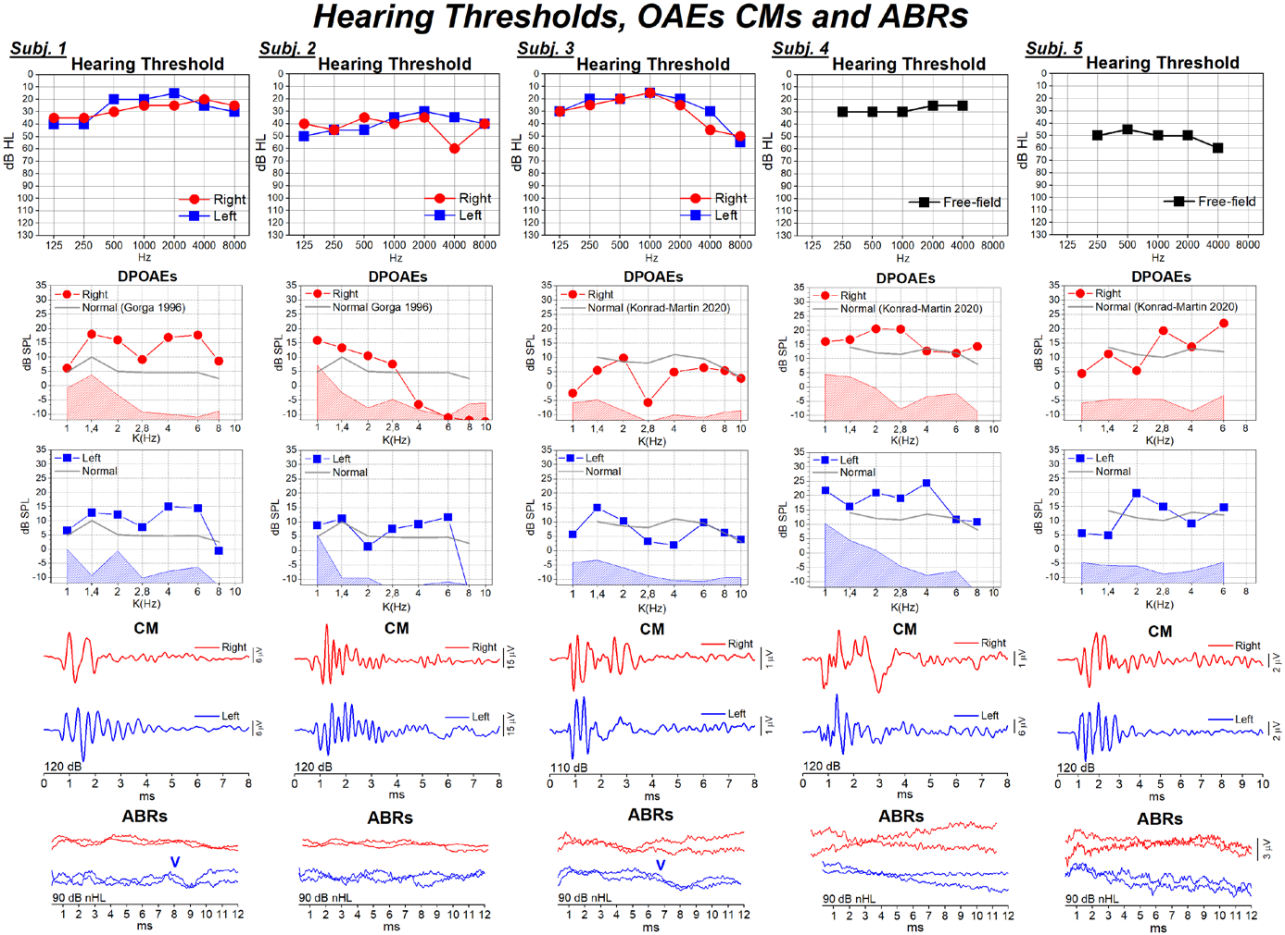
Hearing thresholds, distortion product otoacoustic emissions (DPOAEs), cochlear microphonics (CMs), and auditory brainstem responses (ABRs) in patients with *OTOF*-related mild to moderate hearing loss. Hearing thresholds measured in each ear using conventional audiometry are reported for subjects #1–#3, whereas data obtained in the free field through visual reinforcement audiometry are displayed for subjects #4 and #5. DPOAEs and CMs were recorded from both ears in all subjects. In DPOAE-grams, the continuous gray line indicates mean values obtained in normally hearing peers by Gorga et al. (1997) and Konrad-Martin et al. (2020). CM traces begin 0.5 ms prior to the onset of the CM. ABRs were absent in all but two patients (#1, #3), who showed a small-amplitude delayed Wave V in their left ear.

Hearing thresholds indicated mild sensorineural hearing loss in patients performing conventional audiometry (#1 to #3), and mild (#4) or moderate (#5) hearing loss in the two children submitted to visual reinforcement audiometry in the free field (Table 1; Fig. 3). Tympanometry was normal in all patients (Type A tympanogram). Middle ear acoustic reflexes were detected bilaterally with high thresholds (≥90 dB HL) in three subjects, whereas they were absent in the remaining two subjects (Table 1).

**Fig. 4.**
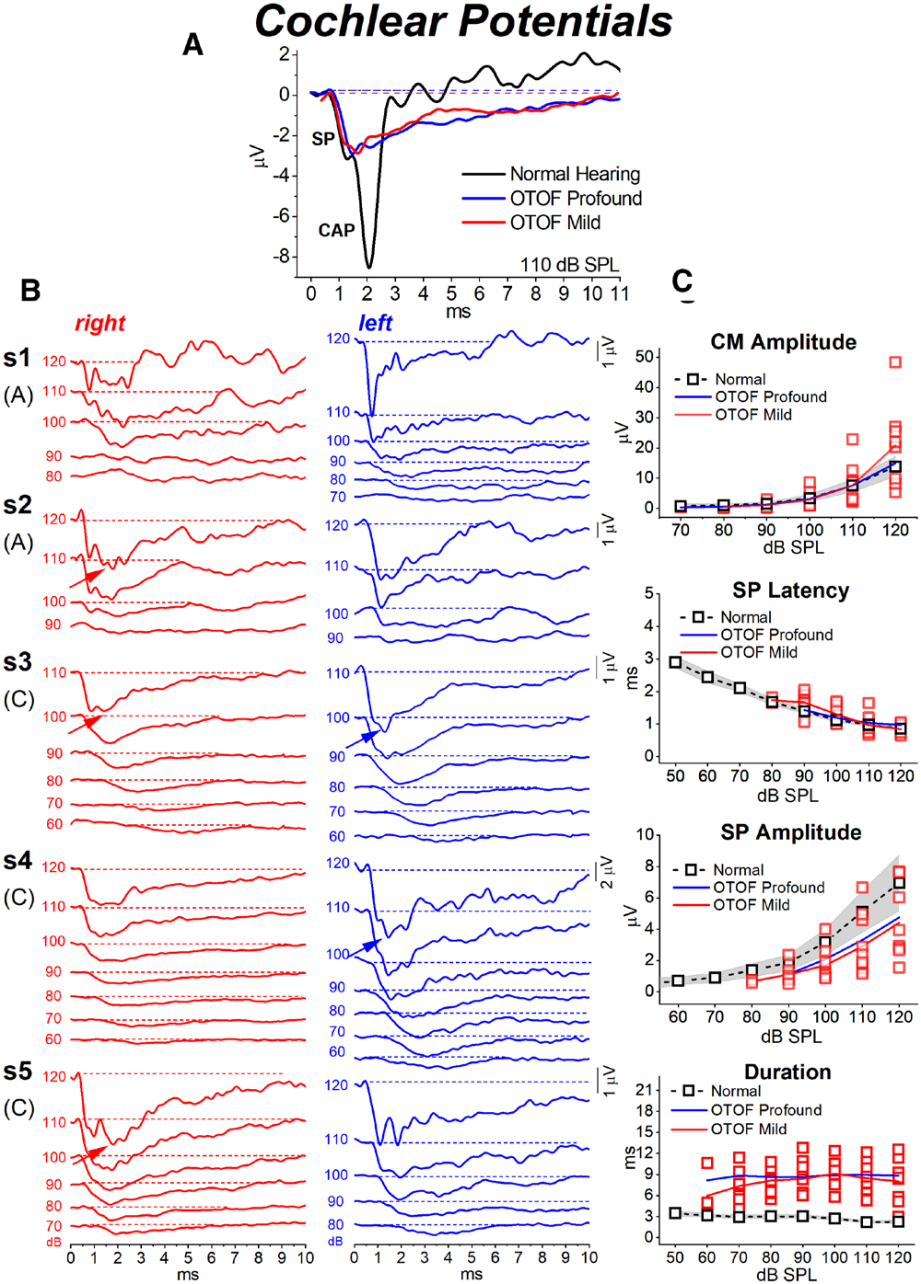
Cochlear potentials recorded from patients with *OTOF*-related mild to moderate hearing loss. A, The electrocochleography (ECochG) waveform obtained after CM cancellation at 110 dB p.e. SPL from one child with *OTOF*-related mild hearing loss (subject #3, left ear) is superimposed on the corresponding traces recorded from two children, one with normal hearing and the other suffering from OTOF-related profound deafness (Santarelli et al., 2015). A synchronous compound action potential (CAP) was recorded only in the normal control, whereas in both OTOF subjects the response begins with the SP followed by a low-amplitude prolonged negative potential. B, The ECochG waveforms obtained at decreasing stimulus intensities are displayed for the five OTOF subjects showing mild to moderate hearing loss. The high-intensity summating potential (SP) was recorded from all patients and it was followed by the prolonged negative potential. In some ears a low-amplitude CAP (arrow) was superimposed at high intensity on the prolonged response. The latter was identified as low as 60–70 dB p.e. SPL in the three children (s3, s4, and s5; C), whereas it was absent at low intensity in the two adults (s1 and s2; A). In all traces time “0” refers to CM onset. Amplitude, latency, and duration of ECochG potentials calculated for the five OTOF patients with mild to moderate hearing loss are reported in the right panel (C). Individual (open red squares) and mean values (red line) are plotted as a function of signal intensity, superimposed on the corresponding mean values (dashed black line) with 95% confidence limits (shadowed areas) calculated for normally hearing controls. The blue line refers to the mean values obtained in a group of 8 children showing *OTOF*-related profound deafness (Santarelli et al. 2015). Compared to normally hearing controls, SP and CM recorded in both groups of OTOF patients showed comparable amplitudes and peak latencies, whereas the duration of the whole ECochG waveforms as measured from the SP onset to return to baseline was significantly increased.

DPOAEs were recorded bilaterally in all patients (Fig. 3). Amplitude was similar to or even larger than that reported for normally hearing subjects of comparable age, except for patients #2 and #3, who showed a reduction of amplitude respectively in the right ear and bilaterally at the mid-frequencies. CM potentials were recorded with normal amplitude in all patients (Figs. 3 and 4).

ABRs were absent in all but two subjects (#1 and #3) who showed a delayed Wave V in their left ear (Fig. 3).

### Electrocochleography

ECochG waveforms obtained after CM cancellation showed remarkable differences in comparison with normally hearing controls. In Figure 4A, ECochG potentials recorded in two children harboring mutations in the *OTOF* gene, one showing mild hearing loss and the other profound deafness, are superimposed on the ECochG waveform recorded in one normally hearing control at an intensity of 110 dB p.e. SPL. In the normal control, the response begins with the receptor SP, which is believed to derive from IHC activation (Durrant et al. 1998). This is followed by the neural CAP, originating from the synchronous activation of auditory fibers innervating the basal portion of the cochlea (Eggermont 1976). In both OTOF children, ECochG waveforms begin with a fast negative deflection, peaking at the same SP peak latency as in the normal control and showing a comparable amplitude. However, the CAP recorded in the normally hearing child was replaced by a low-amplitude negative potential showing a markedly increased duration in comparison with the normal CAP.

ECochG potentials recorded at decreasing intensities in the five OTOF subjects with mild to moderate hearing loss are displayed in Figure 4B. At high intensity (120 to 110 dB p.e. SPL), the responses consisted of the SP followed by the low-amplitude prolonged negative potential. In four patients, a small CAP, peaking approximately at the same CAP peak latency as in normal controls, was superimposed on the prolonged potential (arrows). At intensities lower than 90-100 dB p.e. SPL the ECochG waveforms recorded in children (traces indicated with C in Fig. 4B) mostly consisted of the prolonged potential, which was identifiable as low as 60-70 dB p.e. SPL. In contrast, the two adult patients (traces indicated with A in Fig. 4B) showed no responses at low intensity.

Mean and individual values of amplitudes and latencies of ECochG potentials from the five OTOF patients with mild to moderate hearing loss are plotted as a function of signal intensity in Figure 4C, superimposed on the corresponding mean values calculated for normally hearing controls and for a group of eight profoundly deaf children harboring biallelic mutations in *OTOF* whose ECochG findings have been reported in a previous paper (Santarelli et al. 2015). No significant differences were found in CM amplitude (*F* = 0.07, *p* = 0.935), SP amplitude (*F* = 2.24, *p* = 0.125), and SP peak latency (*F* = 0.75, *p* = 0.482) between groups, although SP amplitudes tended to be smaller in OTOF subjects compared to normally hearing controls. In contrast, the duration of ECochG potentials as measured from SP onset to return to baseline was significantly prolonged in OTOF patients compared to normally hearing controls (*F* = 44.24, *p* = 0.001). No differences in response duration were found between the two groups of OTOF subjects.

### Gap Detection and PTCs

Gap detection was measured in subjects #1 (difference limen = 80 ms) and #2 (difference limen = 24 ms). Values proved remarkably higher in comparison to the scores obtained in a group of 19 normally hearing individuals (mean 5.3 ± 0.35 s.e.m., age range 21 to 35 years).

**Fig. 5.**
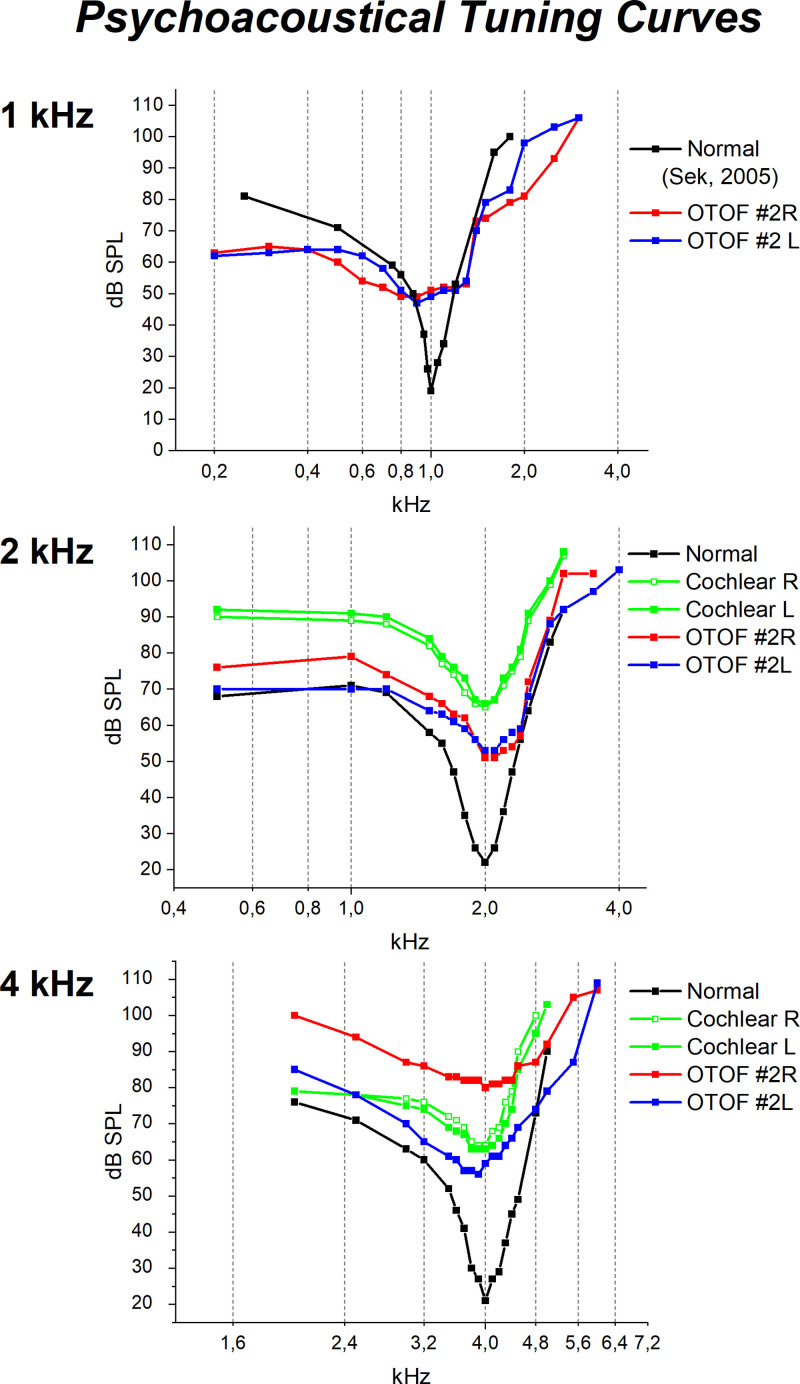
Psychoacoustical tuning curves (PTCs) in one patient (subject #2) showing *OTOF*-related mild hearing loss. PTCs measured in both ears at 2 and 4 kHz are superimposed on the corresponding curves obtained from one normally hearing individual and one subject with cochlear hearing loss (PTA = 45 dB HL). At 1 kHz, PTCs from subject #2 are superimposed on the corresponding curve calculated for a normally hearing subject by Sek et al. (2005). The PTCs measured for the OTOF patient were distorted and showed a truncation of the tip segment in comparison with the normally hearing and hearing-impaired controls. L, left ear; R, right ear.

PTCs were obtained in subject #2 at frequencies of 1, 2, and 4 kHz. These are displayed in Figure 5, superimposed on the corresponding curves measured for two individuals, one with normal hearing and the other with cochlear hearing loss (PTA = 45 dB HL). The curve from the normally hearing control displayed at 1 kHz was from Sek et al. (2005). Compared to the normally hearing controls, PTCs obtained from subject #2 were distorted and showed a truncation of the tip segment. In contrast, the PTC shape was preserved in the hearing-impaired control with cochlear hearing loss.

### Speech Intelligibility and Speech Perception

**Fig. 6.**
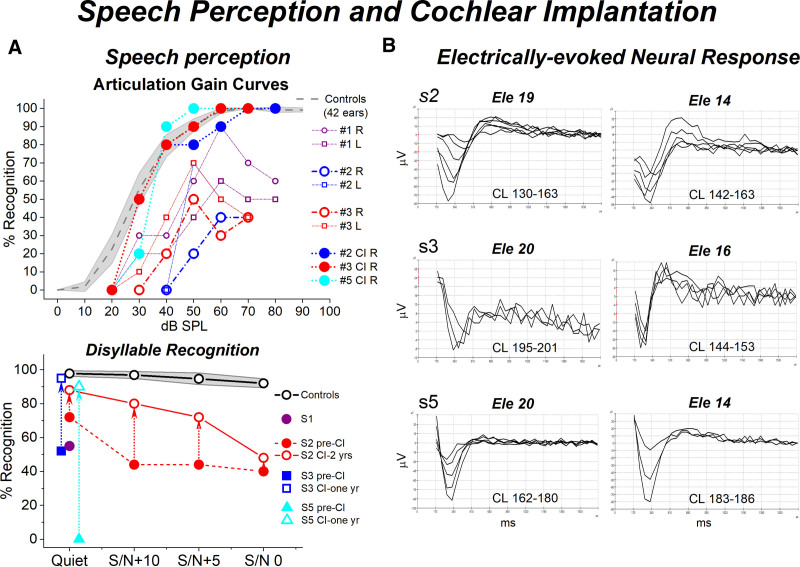
Restoration of speech perception and synchronous activation of auditory nerve after cochlear implantation in *OTOF*-related mild to moderate hearing loss. Articulation gain curves are displayed in the left panel (A, upper part) superimposed on the mean articulation gain curves (dashed gray line) with 95% confidence limits (shadowed area) obtained from a group of 42 ears with mild hearing loss of cochlear origin (PTA = 20–40 dB HL). Compared to the group of controls, OTOF patients showed remarkably lower intelligibility scores. In contrast, the articulation gain curves obtained in the free field after cochlear implantation (CI) in the three cochlear implant recipients showed a remarkable improvement of speech intelligibility scores. In the left panel (A, lower part), the individual scores collected on disyllabic recognition tests are shown for OTOF subjects together with the corresponding mean values obtained for a group of normally hearing individuals. Recognition scores were hugely reduced in OTOF patients compared to controls, but showed a remarkable improvement after cochlear implantation, attaining values of 90 to 95% in all cochlear implant recipients. Recognition scores markedly improved also in the presence of competing noise as evaluated in subject #2. Electrically evoked compound action potentials (E-CAPs) obtained from implanted patients by electrical stimulation through the cochlear implant are displayed in the right panel (B) for three electrode sites at increasing current level (CL). Synchronized neural responses were recorded from all subjects with increasing amplitudes and decreasing peak latency at increasing current level. L, left ear; R, right ear.

Speech intelligibility was tested in cooperative subjects (#1 to #3) (Fig. 6A, upper panel). Individual articulation gain curves were compared to the mean function calculated for a group of adult subjects (42 ears, range 18 to 30 years) showing cochlear hearing loss with comparable PTA levels (20 to 40 dB). In all OTOF subjects, speech intelligibility scores were remarkably lower compared to controls and tended to decrease further at increasing stimulus level.

In accordance with these findings, scores on the disyllabic word recognition test were reduced in the OTOF patients compared to normal hearing controls (the same group as that used for gap detection test) (Fig. 6A, lower panel; Table 1).

### Cochlear Implantation

Articulation gain curves (Fig. 6A, upper panel) were obtained in the free field for subjects #2, #3, and #5 during cochlear implant use. Intelligibility scores showed a remarkable increase compared to preimplant evaluation. The speech detection threshold decreased by 20 dB in both subjects #2 and #3 in comparison with the corresponding values measured in the implanted ear before CI. More importantly, a maximum recognition score of 100% was attained by all subjects at intensities from 50 to 70 dB SPL, which is within the estimated intensity range of conversational speech. In contrast, the maximum recognition scores measured in the implanted ear before CI, ranged from 40 to 50%. Moreover, no distortion was observed in the articulation gain curves during cochlear implant use, whereas the preimplant intelligibility scores decreased at high stimulation intensity (rollover).

In accordance with the findings provided by speech audiometry, disyllable recognition scores significantly improved in all the cochlear implant recipients, attaining values of 90 to 95% within 1 year of cochlear implant use (Fig. 6A lower panel, Table 1). In subject #2, disyllable recognition was also tested in the presence of competing noise at three S/N ratios (+10, +5, and 0). Recognition scores showed an increase of 36 and 28% at S/N +10 and +5 respectively, within 2 years of cochlear implant use.

Sentence recognition improved in all cochlear implant recipients with scores of 90 to 100% within 1 year of implant use (Table 1). Consonant identification improved after CI, although to a lesser extent compared to other speech perception tests possibly due to the involvement in a more difficult perceptual task.

Synchronized neural potentials (electrically e-CAPs) were recorded in all cochlear implant recipients in response to electrical stimulation through the cochlear implant at each electrode location, consistent with restoration of synchronous activation of auditory fibers (Fig. 6B). e-CAP waveforms showed an increase in amplitude and a decrease in peak latency at increasing current levels.

## DISCUSSION

Mutations in *OTOF* cause severe to profound congenital hearing loss in a majority of cases, but there are a few patients whose phenotypes deviate from the general rule. Previous studies have reported mild to moderate hearing loss in patients carrying biallelic mutations in *OTOF* gene for the temperature-sensitive phenotype (Varga et al. 2006; Romanos et al. 2009; Marlin et al. 2010; Wang et al. 2010; Matsunaga et al. 2012; Zhang et al. 2016), in one case of progressive hearing loss (Chiu et al. 2010), and in some siblings of two families with intrafamilial variable severity of hearing loss, which may suggest a progressive phenotype (Yildirim-Baylan et al. 2014; Fedick et al. 2016). In contrast, the subjects included in this study showed a stable phenotype of mild hearing impairment associated with severe deterioration of speech perception in adults and delay of language development in children.

In our previous studies (Santarelli et al. 2009, 2015), transtympanic ECochG was used to define the details of potentials arising in both the cochlea and auditory nerve in profoundly deaf children carrying two mutant alleles in the *OTOF* gene. The findings indicated preservation of receptor potentials, CM and SP, consistent with normal cochlear hair cell activity. In contrast, the CAP originating in normally hearing individuals from synchronous activation of auditory fibers was replaced by a prolonged low-amplitude negative potential, possibly resulting from abnormal activation of auditory terminals due to the impairment of synaptic release. Cochlear implants proved to be effective in restoring hearing sensitivity and speech perception in these children by electrically stimulating the auditory nerve fibers.

ECochG recordings carried out in patients affected by *OTOF*-related mild to moderate hearing loss also showed that the CAP was replaced by a prolonged negative potential, consistent with the lack of synchronous activation of auditory fibers. This activity was indistinguishable from that recorded in *OTOF*-related profound deafness, and it has been interpreted as resulting from abnormal activation of auditory fibers due to impaired multivesicular release. However, differently from OTOF-related profound deafness, the hearing dysfunction affecting these patients is associated with substantial preservation of hearing sensitivity, which has probably contributed to partial development of expressive language. We interpreted these findings as being consistent with selective impairment of vesicle replenishment at the IHCs synapses with relative preservation of synaptic exocytosis. Of note, all mild phenotypes (temperature-sensitive, progressive, and stable) that result from mutations in *OTOF* gene correlate with the presence of at least one missense variant or one in-frame deletion in their associated genotypes (Varga et al. 2006; Romanos et al. 2009; Chiu et al. 2010; Marlin et al. 2010; Wang et al. 2010; Matsunaga et al. 2012; Yildirim-Baylan et al. 2014; Zhang et al. 2016; Fedick et al. 2016; and this article) (Fig. 1), and so partially functional proteins could be synthesized in all these cases. In patients #2 and #3, both alleles carry missense mutations that are novel, that is, not previously reported in subjects with severe or profound DFNB9 hearing impairment (p.Asp1818Tyr, p.Pro1931Leu, and p.Pro1940Arg). Subject #5 carries a frameshift mutation in one allele, but also a novel missense mutation (p.Phe565Ser) in the other allele. In subject #1, we found a missense mutation (p.Pro490Arg) that was previously reported in the homozygous state in a familial case of severe to profound hearing impairment (Al-Wardy et al. 2016). However, the accompanying allele carries a splice-site mutation (c.3127-1G>A) that is predicted to result in an in-frame deletion of only part of the C2D domain (54 residues). Subject #4 was already reported to carry a frameshift mutation in trans with p.Ile1967del, an in-frame deletion of one residue within the only transmembrane domain of otoferlin, which was shown to impair its anchorage to the membrane (Vogl et al. 2016). Diverse missense mutations affecting the functional C2 domains of otoferlin have been reported to cause the severe to profound congenital hearing loss phenotype (Rodríguez-Ballesteros et al. 2008). Only one of the novel missense mutations that we report affects a C2 domain (p.Asp1818Tyr in C2F). However, the mild phenotype that is observed in patient #3 may be attributed to the accompanying allele, p.Pro1931Leu. This mutation, as well as p.Pro1940Arg and p.Phe565Ser, lies outside the known domains, although in conserved regions of the protein. It is remarkable that mutations related to the three different mild phenotypes cluster mainly in two regions: within the C2C domain or very close to its C-terminal end, or in the C-terminal part of the protein (C2E, C2F and transmembrane domains, and their interdomain regions). Establishing correlations between genotypes and these mild phenotypes still needs investigation of new cases. It is also likely that these clinical manifestations may be modulated by diverse modifier effects, as otoferlin interacts with other proteins.

Although the hearing thresholds were not evaluated in each ear for both subjects #4 and #5, we believe that they were symmetrical. Indeed, the threshold of the prolonged ECochG potential recorded in subject #3 was comparable to the hearing thresholds measured through earphones. Moreover, the threshold of the prolonged ECochG component in subjects #4 and #5 was comparable to the hearing thresholds obtained in sound field for both ears. On the basis of these findings, it seems reasonable to admit that hearing thresholds were symmetrical between ears also in subjects performing sound field audiometry.

Interestingly, PTCs measured in subject #2 were distorted and showed a truncation of the tip segment. Ryan et al. (1979) have reported that in chinchillas treated with kanamycin the PTCs showing threshold shifts of 40 to 50 dB were normal in shape and resulted from selective OHC loss. Further elevation in hearing thresholds was associated with distorted PTCs and resulted from both OHC and IHC loss. In agreement with these findings, we found substantial preservation of the shape of PTCs collected from one control subject with cochlear hearing loss who showed a PTA threshold of 45 dB HL. In contrast, OTOF subject #2, although showing comparable or even better hearing thresholds than the control, displayed severely distorted PTCs, consistent with IHC dysfunction.

Another relevant finding of this study consists of the effectiveness of cochlear implants in restoring speech perception in *OTOF*-related mild hearing loss. In our previous article, we reported the benefits of CI in a group of eight profoundly deaf children carrying mutations in both alleles of *OTOF* gene (Santarelli et al. 2015). A positive outcome of using the cochlear implant has also been reported in other studies of profoundly deaf children harboring mutations in *OTOF* (Rodríguez-Ballesteros et al. 2003; Rouillon et al. 2006; Chiu et al. 2010; Runge et al. 2013) and for one patient showing the temperature-sensitive phenotype (Zhang et al. 2016). Nevertheless, this is the first study documenting that cochlear implants constitute a viable therapeutic option in improving speech perception for OTOF patients showing mild hearing impairment. Indeed, speech recognition scores improved remarkably in all cochlear implant recipients within one year of cochlear implant use and progressed thereafter. In agreement with this finding, e-CAP recordings showed the restoration of synchronous activation of auditory fibers in response to the electrical stimulation through the cochlear implant, thus confirming the preservation of auditory fiber excitation and conduction in *OTOF*-related hearing disorders.

In conclusion, mutations of both alleles of the *OTOF* gene may result in alterations of temporal coding of acoustic stimuli with good preservation of hearing sensitivity. Speech perception remarkably improves in response to electrical stimulation through unilateral CI, which remains an effective therapeutic option also in the perspective of future gene therapy possibly targeting the contralateral ear.

## ACKNOWLEDGMENTS

We are greatly indebted to Prof. Roberto Bovo and Dr Franco Trabalzini who performed surgery. We are also grateful to engineer Antonio Selmo who supported us in setting up the recording apparatus.

## References

[R1] Al-Wardy N. M. Al-Kindi M. N. Al-Khabouri M. J. Tamimi Y. Van Camp G. (A novel missense mutation in the C2C domain of otoferlin causes profound hearing impairment in an Omani family with auditory neuropathy. Saudi Med J, 2016). 37, 1068–1075. 27652356 10.15537/smj.2016.10.14967 PMC5075369

[R3] Chiu Y. H. Wu C. C. Lu Y. C. Chen P. J. Lee W. Y. Liu A. Y. Hsu C. J. (Mutations in the OTOF gene in Taiwanese patients with auditory neuropathy. Audiol Neurootol, 2010). 15, 364–374. 20224275 10.1159/000293992

[R4] Cutugno F. Prosser S. Turrini M. (Parole bisillabiche. In Audiometria vocale. 2000). Vol. II (pp 3–13). GN Resound 8135099

[R5] De Filippis A. Cippone P. Leotta M. G. Veronesi E. (Burdo S. (Ed.), Riconoscimento (parole bisillabiche, frasi) nel bambino. Protocollo comune di valutazione in audiologia riabilitativa (PCVRAR) 1997). pp. 46–76 Edizioni CRO

[R37] Durrant J. D. Wang J. Ding D. L. Salvi R. J. (Are inner or outer hair cells the source of summating potentials recorded from the round window? J Acoust Soc Am, 1998). 104, 370–377. 9670530 10.1121/1.423293

[R6] Eggermont J. J. (Keidel W. D. Neff W. D. (Eds.), Electrocochleography. In: Handbook of Sensory Physiology. Auditory System (pp. 1976). Springer.625–706).

[R7] Fedick A. M. Jalas C. Swaroop A. Smouha E. E. Webb B. D. (Identification of a novel pathogenic OTOF variant causative of nonsyndromic hearing loss with high frequency in the Ashkenazi Jewish population. Appl Clin Genet, 2016). 9, 141–146. 27621663 10.2147/TACG.S113828 PMC5012844

[R8] Gorga M. P. Neely S. T. Ohlrich B. Hoover B. Redner J. Peters J. (From laboratory to clinic: a large scale study of distortion product otoacoustic emissions in ears with normal hearing and ears with hearing loss. Ear Hear, 1997). 18, 440–455. 9416447 10.1097/00003446-199712000-00003

[R9] Kluk K. Moore B. C. (Factors affecting psychophysical tuning curves for normally hearing subjects. Hear Res, 2004). 194, 118–134. 15276683 10.1016/j.heares.2004.04.012

[R10] Konrad-Martin D. Knight K. McMillan G. P. Dreisbach L. E. Nelson E. Dille M. (Long-term variability of distortion-product otoacoustic emissions in infants and children and its relation to pediatric ototoxicity monitoring. Ear Hear, 2020). 41, 239–253. 29280917 10.1097/AUD.0000000000000536 PMC6504621

[R11] Marlin S. Feldmann D. Nguyen Y. Rouillon I. Loundon N. Jonard L. Bonnet C. Couderc R. Garabedian E. N. Petit C. Denoyelle F. (Temperature-sensitive auditory neuropathy associated with an otoferlin mutation: Deafening fever! Biochem Biophys Res Commun, 2010). 394, 737–742. 20230791 10.1016/j.bbrc.2010.03.062

[R12] Martini A. Mazzoli M. Kimberling W. (An introduction to the genetics of normal and defective hearing. Ann N Y Acad Sci, 1997). 830, 361–374. 9616696 10.1111/j.1749-6632.1997.tb51908.x

[R13] Matsunaga T. Mutai H. Kunishima S. Namba K. Morimoto N. Shinjo Y. Arimoto Y. Kataoka Y. Shintani T. Morita N. Sugiuchi T. Masuda S. Nakano A. Taiji H. Kaga K. (A prevalent founder mutation and genotype-phenotype correlations of OTOF in Japanese patients with auditory neuropathy. Clin Genet, 2012). 82, 425–432. 22575033 10.1111/j.1399-0004.2012.01897.x

[R14] Pangršič T. Lasarow L. Reuter K. Takago H. Schwander M. Riedel D. Frank T. Tarantino L. M. Bailey J. S. Strenzke N. Brose N. Müller U. Reisinger E. Moser T. (Hearing requires otoferlin-dependent efficient replenishment of synaptic vesicles in hair cells. Nat Neurosci, 2010). 13, 869–876. 20562868 10.1038/nn.2578

[R15] Quaranta A. Arslan E. Babighian G. Filipo R. (Impianto cocleare. Protocolli di selezione e valutazione dei soggetti adulti. Acta Phoniatrica Latina, 1996). 18, 187–265.

[R16] Rodríguez-Ballesteros M. del Castillo F. J. Martín Y. Moreno-Pelayo M. A. Morera C. Prieto F. Marco J. Morant A. Gallo-Terán J. Morales-Angulo C. Navas C. Trinidad G. Tapia M. C. Moreno F. del Castillo I. (Auditory neuropathy in patients carrying mutations in the otoferlin gene (OTOF). Hum Mutat, 2003). 22, 451–456. 14635104 10.1002/humu.10274

[R17] Rodríguez-Ballesteros M. Reynoso R. Olarte M. Villamar M. Morera C. Santarelli R. Arslan E. Medá C. Curet C. Völter C. Sainz-Quevedo M. Castorina P. Ambrosetti U. Berrettini S. Frei K. Tedín S. Smith J. Cruz Tapia M. Cavallé L. Gelvez N. A multicenter study on the prevalence and spectrum of mutations in the otoferlin gene (OTOF) in subjects with nonsyndromic hearing impairment and auditory neuropathy. Hum Mutat, 2008). 29, 823–831. 18381613 10.1002/humu.20708

[R18] Romanos J. Kimura L. Fávero M. L. Izarra F. A. de Mello Auricchio M. T. Batissoco A. C. Lezirovitz K. Abreu-Silva R. S. Mingroni-Netto R. C. (Novel OTOF mutations in Brazilian patients with auditory neuropathy. J Hum Genet, 2009). 54, 382–385. 19461658 10.1038/jhg.2009.45

[R19] Rouillon I. Marcolla A. Roux I. Marlin S. Feldmann D. Couderc R. Jonard L. Petit C. Denoyelle F. Garabédian E. N. Loundon N. (Results of cochlear implantation in two children with mutations in the OTOF gene. Int J Pediatr Otorhinolaryngol, 2006). 70, 689–696. 16226319 10.1016/j.ijporl.2005.09.006

[R20] Roux I. Safieddine S. Nouvian R. Grati M. Simmler M. C. Bahloul A. Perfettini I. Le Gall M. Rostaing P. Hamard G. Triller A. Avan P. Moser T. Petit C. (Otoferlin, defective in a human deafness form, is essential for exocytosis at the auditory ribbon synapse. Cell, 2006). 127, 277–289. 17055430 10.1016/j.cell.2006.08.040

[R21] Runge C. L. Erbe C. B. McNally M. T. Van Dusen C. Friedland D. R. Kwitek A. E. Kerschner J. E. (A novel otoferlin splice-site mutation in siblings with auditory neuropathy spectrum disorder. Audiol Neurootol, 2013). 18, 374–382. 24135434 10.1159/000354978 PMC3877672

[R22] Ryan A. Dallos P. McGee T. (Psychophysical tuning curves and auditory thresholds after hair cell damage in the chinchilla. J Acoust Soc Am, 1979). 66, 370–378. 512200 10.1121/1.383194

[R23] Santarelli R. Starr A. Michalewski H. J. Arslan E. (Neural and receptor cochlear potentials obtained by transtympanic electrocochleography in auditory neuropathy. Clin Neurophysiol, 2008). 119, 1028–1041. 18358774 10.1016/j.clinph.2008.01.018

[R24] Santarelli R. Del Castillo I. Rodríguez-Ballesteros M. Scimemi P. Cama E. Arslan E. Starr A. (Abnormal cochlear potentials from deaf patients with mutations in the otoferlin gene. J Assoc Res Otolaryngol, 2009). 10, 545–556. 19636622 10.1007/s10162-009-0181-z PMC2774414

[R25] Santarelli R. Arslan E., (Celesia G. G. (Ed.), Electrocochleography. Disorders of Peripheral and Central Auditory Processing. In Handbook of Clinical Neurophysiology (pp. 2013). Elsevier.83–113).

[R26] Santarelli R. del Castillo I. Cama E. Scimemi P. Starr A. (Audibility, speech perception and processing of temporal cues in ribbon synaptic disorders due to OTOF mutations. Hear Res, 2015). 330(Pt B), 200–212. 26188103 10.1016/j.heares.2015.07.007

[R27] Sek A. Alcántara J. Moore B. C. Kluk K. Wicher A. (Development of a fast method for determining psychophysical tuning curves. Int J Audiol, 2005). 44, 408–420. 16136791 10.1080/14992020500060800

[R28] Starr A. Sininger Y. Winter M. Derebery M. J. Oba S. Michalewski H. J. (Transient deafness due to temperature-sensitive auditory neuropathy. Ear Hear, 1998). 19, 169–179. 9657592 10.1097/00003446-199806000-00001

[R29] Starr A. Zeng F. G. Michalewski H. J. Moser T. (Dallos P. Oertel D. (Eds.), Perspectives on auditory neuropathy: disorders of inner hair cell, auditory nerve, and their synapse. Audition In The Senses: A Comprehensive Reference. 2008). Elsevier.397–412).

[R30] Varga R. Avenarius M. R. Kelley P. M. Keats B. J. Berlin C. I. Hood L. J. Morlet T. G. Brashears S. M. Starr A. Cohn E. S. Smith R. J. Kimberling W. J. (OTOF mutations revealed by genetic analysis of hearing loss families including a potential temperature sensitive auditory neuropathy allele. J Med Genet, 2006). 43, 576–581. 16371502 10.1136/jmg.2005.038612 PMC2593030

[R31] Vogl C. Panou I. Yamanbaeva G. Wichmann C. Mangosing S. J. Vilardi F. Indzhykulian A. A. Pangršič T. Santarelli R. Rodriguez-Ballesteros M. Weber T. Jung S. Cardenas E. Wu X. Wojcik S. M. Kwan K. Y. Del Castillo I. Schwappach B. Strenzke N. Corey D. P. Tryptophan-rich basic protein (WRB) mediates insertion of the tail-anchored protein otoferlin and is required for hair cell exocytosis and hearing. EMBO J, 2016). 35, 2536–2552. 27458190 10.15252/embj.201593565 PMC5283584

[R32] Wang D. Y. Wang Y. C. Weil D. Zhao Y. L. Rao S. Q. Zong L. Ji Y. B. Liu Q. Li J. Q. Yang H. M. Shen Y. Benedict-Alderfer C. Zheng Q. Y. Petit C. Wang Q. J. (Screening mutations of OTOF gene in Chinese patients with auditory neuropathy, including a familial case of temperature-sensitive auditory neuropathy. BMC Med Genet, 2010). 11, 79. 20504331 10.1186/1471-2350-11-79 PMC2901213

[R33] Yasunaga S. Grati M. Chardenoux S. Smith T. N. Friedman T. B. Lalwani A. K. Wilcox E. R. Petit C. (OTOF encodes multiple long and short isoforms: genetic evidence that the long ones underlie recessive deafness DFNB9. Am J Hum Genet, 2000). 67, 591–600. 10903124 10.1086/303049 PMC1287519

[R34] Yildirim-Baylan M. Bademci G. Duman D. Ozturkmen-Akay H. Tokgoz-Yilmaz S. Tekin M. (Evidence for genotype-phenotype correlation for OTOF mutations. Int J Pediatr Otorhinolaryngol, 2014). 78, 950–953. 24746455 10.1016/j.ijporl.2014.03.022 PMC4066206

[R35] Zeng F. G. Kong Y. Y. Michalewski H. J. Starr A. (Perceptual consequences of disrupted auditory nerve activity. J Neurophysiol, 2005). 93, 3050–3063. 15615831 10.1152/jn.00985.2004

[R36] Zhang Q. Lan L. Shi W. Yu L. Xie L. Y. Xiong F. Zhao C. Li N. Yin Z. Zong L. Guan J. Wang D. Sun W. Wang Q. (Temperature sensitive auditory neuropathy. Hear Res, 2016). 335, 53–63. 26778470 10.1016/j.heares.2016.01.008

